# Why were New World rabbits not domesticated?

**DOI:** 10.1093/af/vfab026

**Published:** 2021-06-19

**Authors:** Andrew D Somerville, Nawa Sugiyama

**Affiliations:** 1 Department of World Languages and Cultures, Iowa State University, Ames, IA, USA; 2 Department of Anthropology, University of California, Riverside, CA, USA

**Keywords:** domestication, human–animal interactions, lagomorphs, rabbit behavior, Teotihuacan

**Implications**

- A greater diversity of rabbit species occurs in North America compared with Europe.
- Archeological evidence demonstrates thousands of years of human–rabbit interactions in both Europe and North America, particularly at the ancient city of Teotihuacan (~AD 1–550) in central Mexico where several studies suggest practices of rabbit management by humans.
- The European rabbit (*Oryctolagus cuniculus*) is the only lagomorph species to have been domesticated by humans.
- This review finds that behavioral differences between European and North American rabbits explain their differential suitability for domestication.

## Introduction

Recent archeological and biological research has produced evidence for thousands of years of interactions between human and leporid (rabbits and hares) populations in both Europe and North America (Canada, United States, and Mexico). Resulting from these relationships, European rabbits (*Oryctolagus cuniculus*) were domesticated in southern France within the last 1500 yr (Carneiro et al., 2011; Irving-Pease et al., 2018), and are now commonly bred around the world for their roles as pets, food, a source of fur, and as laboratory subject animals. North American rabbits, however, were not domesticated in the same manner as their Old World counterpart.

This article explores the factors that may explain this disparity in domestication. We begin by providing a brief evolutionary history of rabbits and hares (family Leporidae). We then review the archeological evidence for human–rabbit interactions in both the Old and New Worlds, with an emphasis on new data from the ancient Mexican city of Teotihuacan, where archeological and chemical evidence suggests the importance of rabbits in the local diet and economy. Finally, we consider the biological and behavioral characteristics of European rabbits and North American cottontails, emphasizing traits that probably influenced their differential domestication outcomes.

## Leporidae

Together with the pika family (Ochotonidae), the Leporidae belong to the order Lagomorpha. The evolution of the leporid family is complex, but the earliest fossils have been found in eastern Asia during the Early to Middle Eocene epoch (~60 to 40 mya) (Ruedas et al., 2018). Leporids spread to North America and throughout the rest of the Old World and experienced a major radiation during the Miocene epoch (~23 to 5 mya) (Lopez-Martinez, 2008; Flynn et al., 2014). This expansion and diversification was likely due to the worldwide spread of C4 grasslands during this global period of cooling and drying (Ge et al., 2013).

Today, the Leporidae family contains 11 genera and 63 species and occupies all major landmasses on earth (Ruedas et al., 2018). Hares belong to a single genus (*Lepus*) with 32 individual species that are native to North America, Europe, Africa, and Asia. The colloquial term of “rabbits” includes 10 distinct genera and 31 species (Smith et al., 2018: 87). Although several hare species are native to Europe, the only extant rabbit species is the European rabbit (*O. cuniculus*). This species includes two subspecies: *O. cuniculus algirus* and *O. cuniculus cuniculus* (Ferrand and Branco, 2007; Lopez-Martinez, 2008). The natural range of *O. c. algirus* is southern and western portion of the Iberian Peninsula, including Spain and Portugal, whereas the range of *O. c. cuniculus* includes the northeast portion of Spain and southern France (Ferrand and Branco, 2007). These populations probably represent centers of refugia during the Last Glacial Maximum. Genetic and protein analyses indicate that the more northern *O. c. cuniculus* subspecies was the population from which domesticated rabbits originated (Branco et al., 2000; Ferrand and Branco, 2007; Carneiro et al., 2011). Indeed, all domesticated rabbit breeds including the English lop, the Angora rabbit, and the New Zealand white rabbit, which is the most commonly used species in biomedical research, are all descendants of this northern Iberian population.

A greater diversity of rabbit species exists in the Americas than in Europe, and they occur in a broader range of environments. Containing 17 species, the most diverse rabbit genus of the New World is *Sylvilagus* (Smith et al., 2018). The most widespread member of the *Sylvilagus* genus and the most common rabbit of North America is the eastern cottontail (*Sylvilagus floridanus*). It occurs from Canada to Venezuela. The two other genera of New World rabbits are the monotypic pygmy rabbits (*Brachylagus idahoensis*), which are found in the western United States, and the monotypic volcano rabbits (*Romerolagus diazi*), which are found in central Mexico.

## Human–Rabbit Interactions in Europe

Archeological and textual evidence demonstrate a long history of human–rabbit interaction in Europe (King and Thompson, 1994; Saña, 2013), particularly in the Iberian Peninsula, which is the native range of *Oryctolagus cuniculus* (Lopez-Martinez, 2008). The first interactions between humans and leporids in Europe began in Spain during the Late Pleistocene epoch (~50 to 30 kya) when Neandertals and Anatomically Modern Humans both hunted rabbits for food and fur (Fa et al., 2013). During the terminal Pleistocene and early Holocene in the Iberian peninsula (i.e., Epipaleolithic, Mesolithic, and Neolithic eras; ~11,500 to 4500 BC), rabbits appear to have been among the most commonly hunted and consumed animals by modern humans, with some faunal assemblages containing over 90% rabbit bones (Saña, 2013).

Throughout the middle and late Holocene, rabbits remained an important prey source for humans across the Iberian Peninsula. Roman sources from around the third century BC document the importation of rabbits to the Italian peninsula and describe the raising of rabbits in managed fields and pens for food and hunting (Flux, 1994). Archeological evidence from the Roman and pre-Roman sites of Ambrussum, Lattara, and Pech Mahoin southern France indicates the presence of rabbit bones, and multivariate analyses of skeletal measurements demonstrate they exhibited a larger size than wild populations, suggesting intentional breeding by humans (Watson, 2019a,b; Watson and Gardeisen, 2019). Additionally, a rabbit bone was recovered from the first to second century AD Fishbourne Roman Palace in Britain (Sykes et al., 2019), suggesting management or at least long-distance trade of rabbits at this time.

Human translocation of breeding populations intensified during the Middle Ages, extending the distribution of European rabbits throughout Europe and beyond after around AD 800 (Flux, 1994; Callou, 2003; Irving-Pease et al., 2018). Archeological sites across large portions of Europe frequently contain associated ruins of large rabbit warrens or pillow mounds (Williamson, 2006; Pelletier et al., 2016), demonstrating human management and the importance of leporids in human subsistence. Clear morphological changes associated with human-directed breeding, however, only occurred during the 18th century AD when rabbit pet-keeping became common (Callou, 2003). Today, rabbits represent one of the most widely dispersed and numerous mammalian domesticates across the globe.

Following Zeder (2015: 3191), we define domestication as “a sustained multigenerational, mutualistic relationship in which one organism assumes a significant degree of influence over the reproduction and care of another organism in order to secure a more predictable supply of a resource of interest….” The timing of when *O. cuniculus* crossed the wild-domesticated boundary is difficult to ascertain, as it was a long-term process rather than a singular historical event (Irving-Pease et al., 2018). Evidence of a strong bottleneck in genetic diversity suggests that a singular population in southern France was domesticated sometime within the last 1500 yr (Carneiro et al., 2011), but morphological changes to the skeleton that distinguish wild from domesticated varieties only appear in the 18th century AD (Callou, 2003). We agree with Larson and Fuller (2014: 127) that the European rabbit (*O. cuniculus*) likely followed the “directed pathway” to domestication, a process that implies the deliberate attempt by humans to domesticate the animal (Zeder, 2012).

## Human–Rabbit Interactions in North America

In North America, rabbits exhibit greater geographic distribution and species diversity than in Europe (Chapman and Litvaitis, 2003). Zooarcheological findings in dry caves of central Mexico containing cottontail rabbits (*Sylvilagus* sp.) indicate their use for food and fur since at least the terminal Pleistocene (Flannery, 1967). After the domestication of plants and the development of farming communities, rabbits remained important sources of food for societies across Canada, the United States, and Mexico. For instance, Lapham et al.’s summary of zooarcheological remains from seven sites in Oaxaca, Mexico, spanning from archaic hunter gatherers campsites (Guila Naquitz, 8700 to 8000 BC) to Early Postclassic cities (Mitla and El Palmillo, AD 1100) demonstrated a consistent pattern of rabbit usage similar to, or even more prevalent than the domesticated dog or turkey (Lapham et al., 2013: Table 3). They argue that rabbits were significant contributors to animal economies at several of the sites they examined, especially at the site of El Palmillo where not only did they contribute between 28% and 39% of the number of identified specimens, they were utilized as food, within rituals, and for their fur, an important component of textile production. Later, in Hernan Cortez’s letters to King George, he described the sale of rabbits at the Aztec marketplace of Tlateloco during the early 16th century AD (Cortés, 1977: 110–114). The best archeological evidence of intensive human–leporid interactions at a single settlement comes from the central Mexican metropolis of Teotihuacan.

## Leporids of Teotihuacan

The ancient city of Teotihuacan, Mexico (AD 1–550) provides one of the best case studies to understand intensive human–leporid interactions in an urban landscape. The city extended over 20 km^2^ and housed a population of about 100,000 inhabitants in orthogonal apartment compounds (Cowgill, 2015). Leporids constituted 23% of the minimum number of individuals (**MNI**) of the Classic Teotihuacan fauna remains analyzed (Sugiyama et al., 2017: Table 3). This total is double the MNI percentage attributed to deer, one of Mesoamerica’s premier large herbivores that was utilized as a standard protein source in other pre-Hispanic urban centers (e.g., Maya sites) (Pohl, 1991; Sharpe et al., 2018; Sugiyama et al., 2018, 2020). In comparison to lagomorph indices (ratio of hares to rabbits) in the southwestern United States, where a large proportion of hares compared to rabbits suggests that large communal hare drives helped sustain human populations (Potter, 1997, 2000), *Lepus/Sylvilagus* ratios at Teotihuacan (0.47) indicate the greater prevalence of rabbits over hares (Sugiyama et al., 2017). One possible explanation is that hares were acquired opportunistically through garden hunting (Linares, 1976), whereas rabbits were not only hunted in the gardens but also captured and opportunistically or extensively kept in the homes. It is particularly noteworthy that a spatial analysis of rabbit and hare remains resulted in a greater density of leporids in the city’s core compared with the periphery, with a particular emphasis on rabbits over hares in various areas along the ceremonial core (Sugiyama, et al., 2017).

The best evidence for rabbit captivity and breeding within the city of Teotihuacan was found within a residential apartment complex in the northeast of the city (N6W3) called Oztoyahualco (Manzanilla, 1993). The archaeological, zooarcheological, and isotopic data suggest household level captive breeding of rabbits not only provided a reliable source of proteins, lipids, and fur to their residents, but was also specialized economic task that generated a surplus to be sold/traded. Archeological indicators of rabbit captivity included several smaller room blocks with high phosphate levels in the floors indicative of the area where the rabbits may have been penned or butchered. Additionally, a stone sculpture of a rabbit found in the central plaza suggests this animal was symbolically and/or economically important to the residents. The zooarcheological report of the compound indicated one of the largest concentrations of leporids from a single context, accounting for 46% of the total faunal assemblage, many of which were obtained from the fill of the aforementioned room blocks with high phosphate levels (Valadez Azúa, 1993). Stable carbon isotope analysis indicates that leporids from Oztoyahualco consumed significantly greater amounts of C4 or CAM plants, such as maize or cactus, than did leporids from other sectors of the city, a pattern that suggests human provisioning of the animals, either in managed fields or within the compound itself (Somerville et al., 2016, 2017). Notably, a diverse mix of leporids was present at Oztoyahualco, including three genera (*Lepus*, *Romerolagus*, and *Sylvilagus*) and six species (*R. diazi*, *S. audubonii*, *S. floridanus*, *S. cunicularius*, *L. callotis*, and *L. californicus*), with the eastern cottontail (*S. floridanus*) being the most commonly represented (Valadez Azúa, 1993: Table 17).

Together, the archeological and isotopic data suggest that humans were provisioning leporids at the Oztoyahualco compound of Teotihuacan and likely producing them for food, fur, and ritual. This emphasis on leporid production and consumption contrasts with the low prevalence of the two domesticated species of Mesoamerica, the dog (*Canis familaris*, 11% MNI) and the turkey (*Meleagris gallopavo*, 6% MNI) (Valadez Azúa, 2003; Manin et al., 2018). Both played a minimal role in dietary practices at Teotihuacan. The presented evidence of rabbits as a predictable source of protein and fat that could be managed at the level of the household or apartment complex.

Evidence of rabbits offered as food for sacrificed animals buried within the Moon and Sun Pyramids at Teotihuacan suggests that rabbits were utilized in state functions. Isotope data confirm that the rabbits found in the stomach contents of ritually sacrificed carnivores, including pumas and eagles, were also fed a diet high in C4 resources. In this way, rabbit production would provide a stable meat source to raise captive carnivores within the city (Sugiyama, et al., 2015). The high concentration of rabbits near the ceremonial core also suggests these predictable resources would have been optimal for use in public feasts and other state functions.

## Comparative Sociality

Despite a far greater diversity of leporid species, over 10 millennia of human–rabbit interactions, and centuries of an intensive relationship at Teotihuacan, cottontail rabbits were not domesticated in North America as they were in Europe. Although European rabbits may have followed the directed pathway to domestication, North American rabbits likely embarked on the commensal or prey pathways, but never reached the final destination. Scholars have long noted that the social behavior of an animal is an important factor in the domestication process (Hale, 1969; Price, 1984; Diamond, 1997; Zeder, 2012). In a summary of the behavioral characteristics favorable for domestication, Zeder (2012: 231) identifies four primary areas that render an animal “preadapted” for domestication. These include 1) *the social structure of the organism*, with favorable traits including large group size, a social hierarchy, and the presence of males within the group; 2) *the sexual behavior of the organism*, with favorable characteristics including a promiscuous mating system, males being dominant, and the signaling of sexual receptivity by females; 3) *parent–young interactions*, with favorable characteristics including social imprinting, females accepting young soon after birth, and precocial offspring; 4) *the nature of the response to humans*, with favorable characteristics including a short flight distance, low reactivity, and the ability to be readily habituated; and 5) *the feeding behavior and habitat choice of the organism*, with favorable characteristics including a generalist feeding strategy, a wide environmental tolerance, and nonshelter seeking. Here we briefly summarize the behavioral ecology of the European rabbit (*O. cuniculus*) and that of the eastern cottontail (*S. floridanus*), which is the most common rabbit of the Americas and was the most abundant species present at Teotihuacan. Because dietary practices, digestive strategies, habitat preference, and response to humans are similar for these species, we focus the discussion on the first three of these behavioral characteristics that “preadapt” an animal for domestication.

### Oryctolagus cuniculus

**Figure 1.**
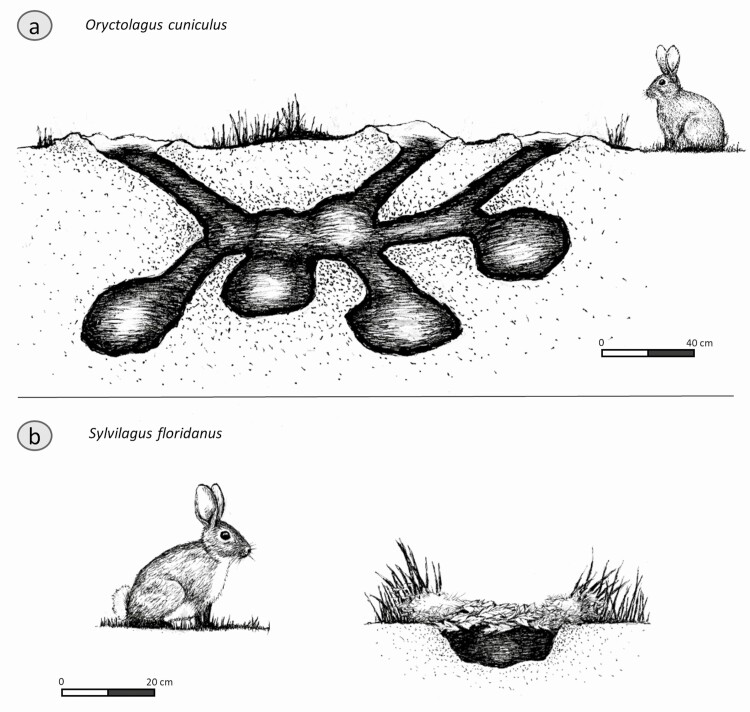
Illustration of the (a) European rabbit and its burrow and (b) the eastern cottontail and its burrow. Illustrations by Nathan Thrailkill.

The natural range of the European rabbit extends across the Iberian Peninsula and varies from woodland to open field habitat. It readily becomes accustomed to human presence and frequently inhabits areas near human settlements. The European rabbit is the only leporid species to form stable social groups under wild conditions (Cowan and Bell, 1986). Groups inhabit multi-entrance burrow and chamber systems known as *warrens* (Pelletier et al., 2016), which are mostly dug by adult females and can reach up to 3 m in depth (Figure 1). Groups are comprised of a dominant male residing and reproducing with one to multiple females and their young offspring (Lockley, 1975). The population of the warren may range from two to 20 adults. In larger communities, subordinate males and juveniles are also present. *O. cuniculus* can be considered a gregarious species. In laboratory settings, rabbits raised in individual cages are generally more stressed, less healthy, and display more pathlogical behaviors, including fur pulling and bar biting, than do group-raised rabbits (DiVincenti and Rehrig, 2016).

The mating system of the European rabbit is primarily polygamous, but the exact social arrangement varies from monogamous pairs, to promiscuity, to harem polygyny (Cowan and Bell, 1986). These differences are ultimately influenced by the spatial availability of suitable burrow space, which determines the distribution of females across the landscape and hence the distribution and intersexual competitive dynamics of males (Myers and Poole, 1959; Mykytowycz, 1959). A ridged dominance hierarchy exists separately for each sex; males compete over access to females, whereas females compete over access to suitable territory for burrow space (Cowan and Bell, 1986).

Females give birth to altricial young and nurse infants only for a few minutes once every 24 hr, and weaning is completed within 3 to 4 wk (Bautista et al., 2008; González-Mariscal et al., 2016). Little parental care exists among *O. cuniculus*, but in an experimental setting, males are known to defend juveniles from antagonistic adult does (Mykytowycz and Dudziński, 1972).

### Sylvilagus floridanus

The eastern cottontail is the most common and widely distributed rabbit species of the Americas, stretching from southern Canada to Venezuela (Smith et al., 2018). They inhabit a variety of ecoregions across their range, but prefer disturbed habitats, such as old fields, shrublands, and generally patchy landscapes with open spaces (Chapman and Litvaitis, 2003; Smith et al., 2018).

The mating system of *S. floridanus* ranges from promiscuous to polygynous. A ridged and linear dominance hierarchy exists among males, principally resulting from male–male competition over access to receptive females with dominant males obtaining more successful copulations (Marsden and Holler, 1964). The establishment of a defended core territory is not a common practice of *S. floridanus*; instead, males chase or dislodge lower ranking males when receptive females are present (Brenner and Flemming, 1979; Smith et al., 2018). Although a separate hierarchy exists among females, it is more flexible and less rigidly enforced, which is likely due to the low overall rate of encounters between females (Chapman and Litvaitis, 2003). Male home ranges are larger than female home ranges as they travel greater distances in search of receptive females and because females restrict their ranges in order to stay near their nests to nurse and defend their young (Trent and Rongstad, 1974). Although daily ranges of male and female individuals often overlap, they do not form into stable social groups (Marsden and Holler, 1964). Males and females are primarily solitary with the exception of the interactions between mothers and offspring, which are themselves infrequent.

Eastern cottontail nests are created by females digging shallow and slanted burrows (~10 to 15 cm deep; Figure 1), which they insulate and conceal with fur and grass (Casteel, 1966; Bruch and Chapman, 1983). The females do not enter the burrows, but crouch above them so the young can nurse from below (Nowak and Walker, 1999). Eastern cottontails are also known to create aboveground shelters within protective brush or use existing burrows created by other species. Contact between mothers and infants is minimal, as the mothers visit the young to nurse only for a few minutes once or twice every 24 hr (Verts et al., 1997).

## Discussion

Although the European rabbit and the eastern cottontail are similar in many ways, including their diet, digestion, and the degree of parental investment for their altricial young, several key differences distinguish these rabbit species. The most significant of these differences concerns the degree of sociality or gregariousness of the rabbits. *O. cuniculus* is a social animal that inhabits large communal warrens, whereas *S. floridanus* is a largely solitary animal. Indeed, Eastern cottontails are difficult to breed in captivity as they often fight when penned together, occasionally resulting in death (Dice, 1929).

Though both European and North American rabbits embarked on pathways to domestication, we suggest the behavioral qualities of European rabbits made them more susceptible to complete the path than eastern cottontails in two primary ways. First, the gregarious nature and ability to form stable social groups allowed European rabbits to be penned by humans and entire breeding populations could be managed within confined areas spaces with a minimal amount of inter-rabbit conflict. Enclosing eastern cottontails would have been more difficult due to their solitary nature and propensity to fight. Secondly, the natural tendency of European rabbits to form spatially clustered breeding groups centered on underground warren systems, would have allowed humans to easily locate, hunt, and eventually enclose and for managed breeding. New World cottontails, on the other hand, are solitary and more diffuse across a landscape making them harder to directly pen and manage.

In addition to their behavioral qualities, the overall diversity of rabbit species in North America may have served as a limiting factor for domestication. The fact that six different leporid species were found among the faunal bones at the Oztoyahualco compound of Teotihuacan indicates that residents practiced mixed acquisition and management strategies of diverse leporid populations rather than managing large breeding colonies of a singular species. Domestication requires a sustained multigenerational relationship with a specific animal population that has restricted gene flow with closely related wild populations (Larson and Fuller, 2014). The diversity of rabbits at Teotihuacan indicates that human residents had more *extensive* than *intensive* relationships with rabbits, a pattern not conducive to domestication. The biodiversity of North American cottontails may have thus acted to discourage the domestication of any singular species, despite direct human provisioning and management, and in spite of the importance of rabbits to human nutrition and culture.

## Conclusion

In this article, we attempted to explain why though both Old World and New World rabbits embarked on pathways to domestication, only Old World rabbits obtained this status. We reviewed the archeological and historical evidence for the antiquity and intensity of human–leporid interactions in both Europe and North America, with an emphasis on new data from the archaeological site of Teotihuacan. We demonstrated that rabbits were dietary staples across large portions of North America and the Iberian Peninsula for many thousands of years. After reviewing the differing behavioral strategies of *O. cuniculus* and *S. floridanus*, we found that the social tendencies of these two species were the factors with the greatest divergence. Although *O. cuniculus* is gregarious and inhabits subterranean communal warrens, *S. floridanus* is solitary and their populations do not spatially cluster. Additionally, the biodiversity of rabbit species in North America encouraged humans to engage in extensive relationships with multiple leporid taxa rather than an intensive relationship with a singular rabbit species, as had occurred with *O. cuniculus* in Europe. We suggest that these factors made the European rabbit a more likely candidate for domestication than eastern cottontails.

Finally, the parallels observed in the human–rabbit relationships in Europe and North America challenge us to reconsider the utility of the concept of domestication itself. For thousands of years and in both Europe and North America humans hunted, traded, and managed rabbits in captivity, but one rabbit population ultimately became the focus of intensive directed breeding by humans in southern France. Although all domesticated breeds today can be traced back to this small population, the archeological record demonstrates the rich history of human–rabbit interactions in Europe and North America. The observation that humans were moving, managing, and feeding populations of wild rabbits in multiple areas around the globe prior to the domestication of the European rabbit suggests that the binary distinction between wild and domesticated may fail to capture the complexities of many human–animal relationships.

## About the Authors

**Andrew Somerville** is an assistant professor of anthropology in the Department of World Languages and Cultures at Iowa State University (ISU). He received a PhD in Anthropology from the University of California, San Diego, and spent a year as a UCMEXUS postdoctoral scholar at the National Autonomous University of Mexico in Mexico City. At ISU, he directs the Paleoecology Laboratory and the Graduate Certificate Program in Forensic Sciences. His research focuses on understanding social-environmental dynamics of change in the pre-Hispanic Americas.

**Nawa Sugiyama** is an assistant professor at the University of California, Riverside. She has applied zooarchaeological and bone isotope methodologies to understand New World animal management strategies at Teotihuacan, Mexico, the Maya site of Copan, Honduras, and in various sites in Panama. Her research at Teotihuacan has covered topics pertaining to the construction of ritualized landscapes, human–animal interactions, and urban foodways. She is currently codirector of the Project Plaza of the Columns Complex.
